# Ghost Rates Under the No Surprises Act: How Insurer Calculations Artificially Lower the Qualifying Payment Amount

**DOI:** 10.7759/cureus.115212

**Published:** 2026-08-26

**Authors:** Andrew M Klapper, Anthony N Dardano, Michael Risin, Taylor Florio, Martha Denavea

**Affiliations:** 1 Plastic and Reconstructive Surgery, Delray Medical Center, Delray Beach, USA; 2 Nursing, Revenue Cycle, and Clinical Documentation, Floridian Institute of Plastic Surgery, Delray Beach, USA

**Keywords:** fifth circuit, ghost rates, health insurance transparency, independent dispute resolution, insurer payment benchmarks, no surprises act, out-of-network physicians, qualifying payment amount

## Abstract

The United States No Surprises Act removed patients from many payment disputes between nonparticipating clinicians and insurers while giving substantial influence to the qualifying payment amount (QPA) calculated by the insurer.

This editorial examines whether a federally authorized payment benchmark calculated by the party responsible for payment, using data unavailable to the opposing party, can satisfy basic requirements of legitimacy, reproducibility, and accountability. On August 11, 2026, the en banc United States Court of Appeals for the Fifth Circuit held that the challenged federal methodology was inconsistent with the statute because it required the inclusion of nonnegotiated “ghost rates.” The court concluded that these rates produced artificially low QPAs. Federal independent dispute resolution data published by the Centers for Medicare & Medicaid Services for the third and fourth quarters of 2025 show that providers prevailed in 84% of determinations that were fully contested and did not involve default, and that selected offers exceeded the QPA in approximately 87% of reported comparisons. These results do not establish that every provider offer was reasonable or that ghost rates explain every award. They demonstrate the need for transparent benchmark construction. We recommend disclosure of QPA methodology and rate populations, confidential access by independent auditors to rate-level data, and independent reproduction of disputed QPAs.

## Editorial

Public discussion of the No Surprises Act has often treated anomalous physician bills and provider use of arbitration as evidence of systemic abuse. By contrast, a federal appellate decision concluding that the qualifying payment amount (QPA) methodology produced “artificially low” benchmarks has largely been treated as an administrative law dispute. That asymmetry deserves comparable scrutiny.

On August 11, 2026, the en banc United States Court of Appeals for the Fifth Circuit held in Texas Medical Association v. United States Department of Health and Human Services that the challenged federal rules were inconsistent with the No Surprises Act because they required insurers to include nonnegotiated “ghost rates” in QPA calculations. The court vacated the portions of the rule requiring their inclusion and stated that the resulting QPAs were “artificially low” and had “upended” the federal dispute resolution process [1]. The implications extend beyond administrative procedure to the integrity of the payment benchmark itself.

The United States No Surprises Act protects patients from many out-of-network payment disputes and establishes a federal independent dispute resolution (IDR) process between health plans and nonparticipating providers. It defines the QPA, in relevant part, by reference to the plan or issuer’s median contracted rates for the same or similar item or service [2]. The QPA influences patient cost sharing, initial payment, negotiation, and arbitration.

In the disputes addressed here, the nonparticipating physician has no network agreement with the insurer governing payment for the disputed service. Nevertheless, the QPA draws from contracts between that insurer and other providers. In practical effect, rates from agreements the physician did not sign and cannot inspect become a reference point against which the physician’s payment offer is judged.

Although the QPA is presented as an objective numerical benchmark, it is calculated by the plan or issuer using its own contracted-rate data. The insurer controls the underlying contracts and data, calculates the benchmark, makes the initial payment, and may then invoke that benchmark as evidence in the payment dispute. The nonparticipating physician generally cannot inspect the complete rate population or independently reproduce the calculation. We identify this lack of reciprocal access as the central structural problem.

Ghost rates illustrate the consequences of that information imbalance. Provider contracts may contain extensive default fee schedules, including rates for services the provider does not perform and did not meaningfully negotiate. No service is rendered, and no claim is paid at such a rate. Under the challenged rules, however, a nominal nonzero entry, including a $1 rate, could enter the median calculation as a contracted rate.

The Fifth Circuit cited a survey finding that 68% of primary care professionals had contracts containing rates for services performed fewer than twice annually and that 57% of respondents had contracts containing rates for services they never provide [1]. These figures describe the survey respondents’ contracts; they are not estimates of the percentage of all QPA inputs composed of ghost rates.

Six judges dissented from the majority’s interpretation of the ghost-rate provision, reasoning that “provided” could mean made available and that utilization screening would be impractical within the QPA’s fixed 2019 framework. The majority rejected that interpretation and vacated the portions of the July Rule requiring inclusion of ghost rates. Separately, the court upheld the exclusion of single-case air ambulance agreements under a different statutory provision [1].

In our view, treating unnegotiated rates for services never rendered as observations of market price is not price discovery. It produces a purported market price from transactions that did not occur.

A Health Affairs analysis describes the use of predictive artificial intelligence in postpayment insurance audits, while Optum markets payment-integrity systems designed to identify and recover improper payments [3,4]. These sources do not establish that the same tools could immediately perform complete QPA utilization validation. They do demonstrate that insurers maintain sophisticated infrastructure for examining claims and payment data. We interpret the contrast as an incentive asymmetry: substantial technological scrutiny is directed toward recovering payments, while the inputs determining what insurers initially owe have remained unavailable for independent reproduction.

The narrative's double standard

We argue that the prevailing public narrative applies different presumptions to the two sides of the payment dispute. Physician outlier charges and provider arbitration filings are frequently treated as evidence of systemic abuse, while QPAs calculated by insurers have often been treated as objective market measures despite the opposing party’s inability to reproduce them.

If physicians used nominal $1 rates for services never rendered, withheld the underlying dataset, and relied on those figures to increase what another party owed, the methodology would receive immediate scrutiny. The same scrutiny should apply when the direction of the financial effect is reversed. Reduced physician payment does not make an unreliable benchmark more legitimate.

Physicians and hospitals experience the immediate financial effects, making the harm less publicly visible. We are concerned that sustained artificial underpayment may weaken network participation and reduce the availability of emergency and hospital-based specialists. Although patients are removed from the disputed bill, they may remain exposed to downstream deterioration in access to care.

Federal IDR data provide additional context. Because providers choose which eligible disputes to pursue, these results cannot be generalized to all out-of-network claims or used alone to establish that the QPA is invalid. Providers do not, however, control which offers an independent arbitrator selects. During the third and fourth quarters of 2025, providers prevailed in 84% of fully contested, nondefault determinations, and selected offers exceeded the QPA in approximately 87% of reported comparisons [5]. These figures do not prove that every provider offer was reasonable or that every insurer acted improperly. They show that, among disputes reaching contested adjudication, the QPA frequently failed to predict the selected payment. The Fifth Circuit identified artificially low ghost-rate inputs as one defect capable of contributing to that divergence [1]. The billing benchmark asymmetry and key supporting evidence are presented in Table 1.

**Table 1:** The billing benchmark asymmetry and key evidence. Sources: Statutory and court findings, including the survey reported in the court’s opinion [1,2]; Health Affairs analysis and insurer materials [3,4]; federal IDR data for the third and fourth quarters of 2025 [5]. The public narrative and policy implications represent the authors’ interpretation of the cited evidence. Abbreviations: IDR, independent dispute resolution; QPA, qualifying payment amount.

Dimension	Physician/provider side	Insurer/QPA side
Underlying information	Claim and billed services disclosed to payer	Contracted rate population unavailable to noncontracting physician
Technology and scrutiny	Claims reviewed for payment errors	Postpayment analytics used; QPA inputs not reproducible by noncontracting physicians
Public narrative	Outlier bills framed as systemic abuse	Ghost-rate benchmark received little comparable mainstream scrutiny
IDR outcomes	Provider offers selected in 84% of fully contested determinations without default	Selected offers exceeded the QPA in approximately 87% of comparisons
Ghost rate prevalence	57% of survey respondents reported contracts containing rates for services they never provide [1]	Challenged rules permitted nonzero ghost rates to enter QPA calculations [1]
Legal result	Individual payment disputes remain resolved case by case	Court vacated portions of the July Rule requiring inclusion of ghost rates [1]

No secret benchmarks: who audits the QPA?

Under the challenged framework, the plan or issuer calculated a benchmark affecting its own payment obligation using contract data unavailable to the nonparticipating provider. The Fifth Circuit adjudicated the inclusion of ghost rates. In our view, the decision also exposes a broader structural conflict created when an interested party controls a federally consequential benchmark that the opposing party cannot reproduce.

The court did not determine that other QPA inputs, such as specialty classifications, geographic definitions, plan aggregation, inflation adjustments, modifiers, bundled services, or retrospective compensation, were defective. Its decision nevertheless demonstrates why those inputs should be independently auditable. Some discrepancies may be errors; others may have systematic financial effects. Without independent access, neither possibility can be adequately tested.

We recommend a disclosure and audit framework that protects legitimate trade secrets without shielding benchmark construction from verification. Each disputed QPA should disclose its applicable specialty, geographic region, data year, aggregation methodology, treatment of non-fee-for-service compensation, total rate population, and number of rates associated with actual claims. An independent regulator or qualified auditor should receive confidential rate-level access and be able to reproduce the reported median. Individual contract terms need not be disclosed publicly.

Transparency is not an administrative embellishment. It is the central policy lesson of this decision. A benchmark deserves governmental authority only when its inputs can be examined by an independent body, its methodology reproduced, and its discretionary classifications meaningfully challenged.

The Fifth Circuit’s holding does not establish bad faith by every insurer or invalidate every QPA. It establishes that the challenged federal methodology required the inclusion of nonnegotiated rates and, in the court’s analysis, produced artificially low benchmarks. The lesson is not that patient protections should be weakened. It is that patient protection and valid payment measurement must coexist. The federal agencies administering the No Surprises Act should require independent reproduction of every disputed QPA before that benchmark is given evidentiary weight in federal IDR.

## References

[1] (2026). Texas Medical Association v. United States Department of Health and Human Services, No. 23-40605. Texas Medical Association v. United States Department of Health.

[2] (2026). Preventing surprise medical bills. https://www.govinfo.gov/content/pkg/USCODE-2024-title42/html/USCODE-2024-title42-chap6A-subchapXXV-partD-sec300gg-111.htm.

[3] Mello MM, Trotsyuk AA, Mahamadou AJ, Char D (2026). The AI arms race in health insurance utilization review: promises of efficiency and risks of supercharged flaws. Health Aff (Millwood).

[4] (2026). Payment integrity solutions for health plans. https://business.optum.com/en/operations-technology/payment-integrity.html.

[5] (2026). Federal independent dispute resolution public use files: supplemental background on 2025 Q3 and Q4. https://www.cms.gov/priorities/innovation/data-and-reports/2026/federal-idr-supplemental-background-2025-q3-2025-q4.

